# Diplomate in Medical Laboratory Immunology Certification Examination: A New Chapter for Medical Laboratory Immunology

**DOI:** 10.4049/immunohorizons.2300030

**Published:** 2023-08-28

**Authors:** Aaruni Khanolkar, Amy Spiczka, Tracey L. Bonfield, Thomas S. Alexander, John L. Schmitz, Diana Boras, Karen Fong, Sarada L. Nandiwada, Gerald C. Miller, Anne E. Tebo

**Affiliations:** * Department of Pathology, Ann and Robert H. Lurie Children’s Hospital of Chicago, Chicago, IL; † Department of Pathology, Northwestern University, Chicago, IL; ‡ American Society for Clinical Pathology, Chicago, IL; § Department of Genomics and Genome Sciences & Pediatrics, Case Western Reserve University, Cleveland, OH; ¶ Incite Health, Inc., Doylestown, PA; ‖ Department of Pathology and Laboratory Medicine, University of North Carolina at Chapel Hill, Chapel Hill, NC; # Department of Pediatrics, Section of Allergy & Immunology, Texas Children’s Hospital, Baylor College of Medicine, Houston, TX; **Pathology Laboratory Associates, Tulsa, OK; †† Division of Clinical Biochemistry and Immunology, Department of Laboratory Medicine and Pathology, Mayo Clinic, Rochester, MN

## Abstract

It is indeed a privilege to be an immunologist in what is arguably the golden age of immunology. From astounding advances in fundamental knowledge to groundbreaking immunotherapeutic offerings, immunology has carved out an enviable niche for itself in basic science and clinical medicine. The need and the vital importance of appropriate education, training, and certification in clinical immunology was recognized by the World Health Organization as far back as 1972. In the United States, Ph.D. scientists with board certification in medical laboratory immunology have served as directors of high-complexity Clinical Laboratory Improvement Amendments– and College of American Pathologists–certified clinical immunology laboratories since 1977. From 1977 to 2017, board certification for medical laboratory immunology was administered by the American Society for Microbiology through the American Board of Medical Laboratory Immunology examination. The American Board of Medical Laboratory Immunology examination was phased out in 2017, and in the fall of 2019, the American Society for Clinical Pathology (ASCP) Board of Certification (BOC) examination committee took on the responsibility of developing a new doctoral-level certification examination for medical laboratory immunology. This transition to the ASCP BOC represents a well-deserved and much-needed recognition of the rapid advances in and the highly specialized nature of medical laboratory immunology and its ever-increasing relevance to patient care. This new ASCP BOC certification is called the Diplomate in Medical Laboratory Immunology, and, as of April 1, 2023, it is now available to potential examinees. In this report, we describe the examination, eligibility routes, and potential career pathways for successful diplomates.

## Introduction

It has been said that every successful venture relies on three key pillars: people, product, and process. This notion envisages great leaders supporting smart and talented people to generate the right product and using the right processes to get the product to where it needs to be (1). A half-century ago, the World Health Organization and some other visionary leaders realized the far-reaching potential of what was at that time a nascent subspecialty called “clinical immunology” (2–9). This led to the establishment of an early framework and structure aimed at designing the appropriate educational and training curriculum for and certification in clinical immunology (10). Medical laboratory immunology was a critical component of this framework, and this field has greatly benefited from the pioneering and visionary leadership of John Fahey and Noel Rose (10–12). In 1999, John Fahey penned a prescient narrative on the future of medical laboratory immunology in which he described the evolution of the role of the clinical immunology laboratory from primarily focusing on infectious disease serology and performing CD4 T cell counts for patients infected with HIV to highly sophisticated and esoteric evaluations of cellular immune function, clinically validated novel lymphocyte subsets, and the assessment of outcomes of immunotherapeutic interventions to either stimulate or dampen immune responses (12). Noel Rose was a prominent and early advocate of the American Board of Medical Laboratory Immunology (ABMLI), guiding its evolution from an embryonic concept to a reality that stood the test of time (1977–2017). Commensurate with the rapid pace of advances in immunology, clinical immunology and clinical laboratory immunology also continue to expand their footprint across the clinical medicine firmament. As previously foreseen, the testing portfolio of a full-service, high-complexity clinical immunology laboratory now extends far beyond microbial serology and CD4 T cell count measurements for patients with HIV, and thus, consequently, by 2017, the association of the ABMLI with the American Society for Microbiology (ASM) had run its natural course (12).

Since the turn of the 21st century, immunotherapeutic options, including those that can often shift treatment paradigms for diseases once deemed terminal, have been and continue to be developed on a regular basis (13, 14). Another major development that has had a profound impact on the clinical immunology laboratory has been the rapid growth of clinical genomics and molecular diagnostics (15). The vast array of highly sophisticated tools that are now available to interrogate the human genome and the host immune system has facilitated a close alignment between these two clinical laboratory disciplines. Consequently, advances in both genomics and immunology often complement and catalyze each other. A good example of this symbiosis is clearly evident in the arena of inborn errors of immunity (IEIs). Within the past decade, the number of IEIs has expanded from just over 250 to almost 500 (16). Once a genetic aberration is identified in a patient sample, the diagnostic immunology laboratory plays a critical role in confirming the deficiency or overexpression and the loss- or gain-of-function phenotype of the affected gene product (17–22). Alternatively, on occasion, the rapid identification of a potential defect at the cellular level in a diagnostic immunology laboratory can guide the targeted downstream assessment of the molecular defect, thus preventing an unnecessary and expensive genetic “fishing expedition” (23–27).

The core responsibilities of a board-certified medical immunology laboratory director have been outlined in a previous publication (28). An essential attribute of directorship is also the ability to keep one’s finger on the pulse of both the academic and economic aspects of running a clinical laboratory. Given the rapid profusion of knowledge in immunology, the desire to develop and validate new clinical tests must be viewed carefully through the lens of both the ground-level economics and the strategic value of implementing new tests. For instance, at the very least, the laboratory director has to consider the feasibility and the logistics (e.g., the expected test volumes, availability of manpower resources, specimen transport and stability issues, expectations of clinicians regarding the result turnaround times). On one hand, if a test is designed to diagnose a rare clinical condition, a balanced decision is needed to weigh the cost of developing and maintaining the test (for mandatory College of American Pathologists [CAP] and alternate proficiency testing) against the relatively low clinical demand for the test. On the other hand, bringing a highly specialized test in-house for a rare clinical entity that is performed by only a few select laboratories can elevate and enhance the profile and the national reputation of the laboratory (strategic value). Hence, not every decision needs to be measured against the weight of a coin, and appropriate training and experience can help make these kinds of judgment calls.

From the time the ABMLI examination was phased out in 2017, a group of ABMLI diplomates broached the idea of a new doctoral-level certification examination in immunology with the American Society for Clinical Pathology (ASCP), which eventually resulted in the ASCP Board of Certification (BOC) taking on the responsibility for developing the Diplomate in Medical Laboratory Immunology (DMLI) certification. The ASCP BOC has been in operation since 1928, and it is the industry-leading benchmark for certifying individuals who aim for a career in the clinical/medical laboratory field (29). In conjunction with the long-standing expertise in certification examination development and validation as well as other pertinent resources available within the ASCP BOC, the DMLI examination committee has developed this new DMLI certification examination (30).

### An introduction to the clinical laboratory

A major difference between research and clinical laboratories is that all clinical laboratories in the United States (with the exception of clinical laboratories in exempt states: New York and Washington) are regulated by the Centers for Medicare and Medicaid Services (CMS) through the Clinical Laboratory Improvement Amendments (CLIA) of 1988 (31–33). Another significant difference is that the charges for testing performed in clinical laboratories are billed to the patient (the insured) and are also reimbursed by the CMS for those covered by Medicare and Medicaid (34).

#### CLIA certification

CLIA entails a broad and in-depth regulatory framework of federal standards that apply to all laboratories under its purview; that test human specimens for health assessment; and to diagnose, prevent, or treat disease (31). The overarching objective of the CLIA program is to establish quality benchmarks for clinical laboratory testing (31). The CLIA program is implemented by the Division of Clinical Laboratory Improvement and Quality that operates under the aegis of the Quality, Safety and Oversight Group that exists within the Center for Clinical Standards and Quality at CMS (31). The CAP is one of seven approved accreditation organizations with deeming authority under the CLIA program, and it is widely considered one of the most rigorous accreditation programs for clinical laboratories (33, 35, 36). Broadly speaking, and from a practical standpoint of the laboratory, CLIA and CAP rules emphasize a rigorous and stringent process relating to method validation and assay performance characteristics for the tests performed in clinical laboratories, as well as for the credential requirements (certification and/or licensure) of the personnel directing, supervising, and performing the testing (35). The test method validation must include detailed documentation listing the intended use of the test, the type of analyte (measurand), and requirements for performance characteristics (accuracy or validity: sensitivity and specificity or method comparison, precision, limit of detection, analytical measuring range, linearity, reference intervals, analytical specificity, and analytical sensitivity) (37–41). Quality control (QC) and quality assessment (QA) are of paramount importance, and QC parameters for each assay have to be established, observed, and meticulously documented on a daily basis and must be readily available for review during unannounced inspections (42–44).

#### Inspections of clinical laboratories

Inspections constitute an integral aspect of ensuring compliance with federal standards and thus public trust in the quality of the testing performed in clinical laboratories (44).

Unannounced inspections of clinical laboratories are performed biennially by the CMS-approved accrediting bodies, such as the CAP and The Joint Commission (44, 45). Other agencies, such as the Occupational Safety and Health Administration and the Commission on Laboratory Accreditation, can also inspect clinical laboratories without providing advance notice (43). Unannounced inspections can also be carried out by the state health departments on behalf of the CMS in response to anonymous complaints made in good faith that raise concerns about the quality of the testing being performed in the laboratory arising from perceived violations of CLIA rules and regulations (43, 44).

During the inspection, the team of inspectors goes through a checklist of items pertinent to each specific laboratory and/or laboratory section to ascertain compliance or noncompliance (46). Briefly, this checklist of items includes a fairly exhaustive review of test manuals, procedures (including discontinued procedures), reagents, calibration, QC, proficiency testing, instrumentation installation and service records, kit manufacturer’s user guides, and laboratory personnel files (certifications; licensure; continuing education [CE] transcripts; and, if needed for the job, appropriate immunization records stored in a confidential file) (43, 46). The inspectors also have access to the list of deficiencies noted in the previous inspection cycle for each laboratory; hence, documentation detailing the corrective actions taken to address those deficiencies is also reviewed (43, 44, 46). Deficiencies noted by the inspectors can be corrected on-site during the inspection itself (if very minor) or within 30 d of the conclusion of the inspection (44). In the latter instance, documentation detailing a corrective action plan as well as evidence of immediate implementation of the corrective action taken has to be provided to the appropriate accrediting agency (44). Major infractions such as violations that put patients in “immediate jeopardy” can result in principal sanctions (suspension, limitation, revocation of the CLIA certificate) and/or alternative sanctions (a directed plan of correction, state on-site monitoring, civil money penalties), civil suits, and criminal sanctions (47–51).

#### Clinical immunology laboratory

A detailed account of the discipline of clinical laboratory immunology and the critical role that Ph.D. trained directors play in clinical laboratories has been reported previously (28, 35). Briefly, clinical immunology laboratories perform testing that supports the clinical services covering immune-deficiency diseases, infectious diseases, autoimmunity, hematologic malignancies, allergy, and transplantation. Depending on whether the laboratory is based in a pediatric or an adult patient setting, the menu of testing options offered by the laboratory can vary and can be tailored to serve the specific needs of the patient population covered. For instance, immunology laboratories based at some children’s hospitals affiliated with large academic medical centers offer a fairly comprehensive panel of high-complexity cellular immunology tests (including tests that assess immune function) aimed at diagnosing IEIs, whereas laboratories in hospitals that primarily serve adult patients often have a very active protein immunology section offering high-throughput serum protein electrophoresis and immunofixation assays for diagnosing monoclonal gammopathies (16, 52). Furthermore, given the rapid evolution that occurs within the immune compartment as one progresses from the neonatal stage to infancy and then early childhood to adolescence and beyond, the pediatric immunology laboratory is required to perform due diligence to carefully establish multiple age range–associated reference ranges for cellular and humoral immune response testing, as opposed to an immunology laboratory primarily serving adult patients, where a single adult age group–specific reference range is usually employed (53).

### The DMLI examination and the educational and training requirements for DMLI certification

**FIGURE 1.**
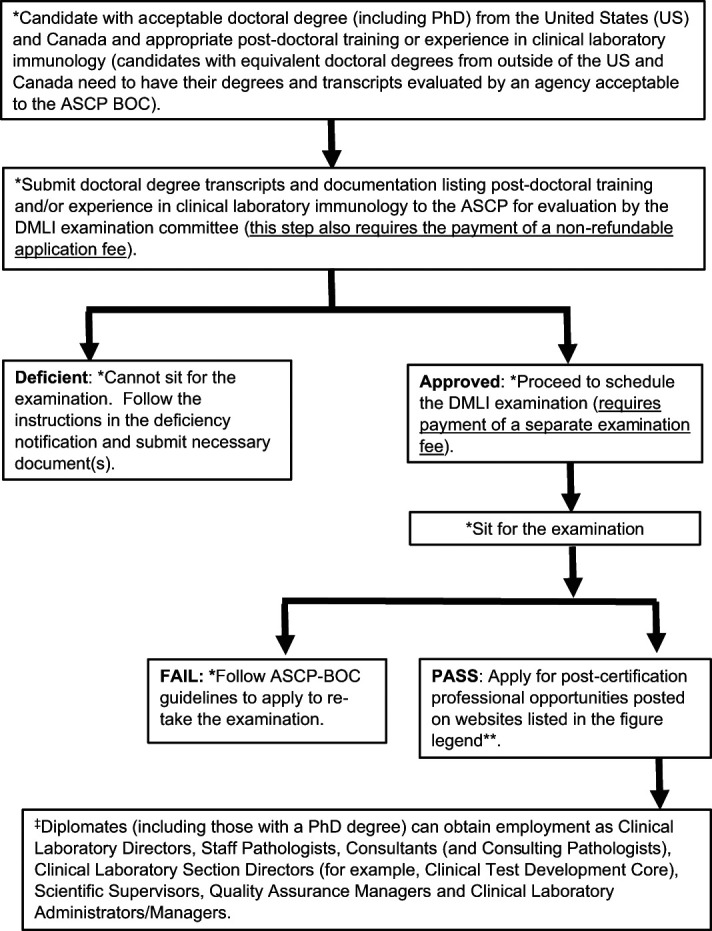
Schematic representation of the pathway to and beyond DMLI certification. *The DMLI certification examination information listed on the ASCP BOC Web site is a living document. Prospective applicants should refer to this document for up-to-date information relating to the certification process. **Clinical laboratory–related jobs are posted on the Web sites of the following organizations: Pathology Outlines (https://www.pathologyoutlines.com/jobs), USAJOBS (the federal government’s official employment site; this site lists positions available at Veterans Administration hospitals), AMLI: Association of Medical Laboratory Immunologists (https://amli.org), and CAP (pathology jobs, CAP Career Center [careerwebsite.com]). Job postings related to the clinical laboratory may also be listed at the Web sites of the following professional societies: ISAC: International Society for Advancement of Cytometry; FOCIS: Federation of Clinical Immunology Societies; ICCS: International Clinical Cytometry Society, as well as the Web sites for companies such as Indeed and Glassdoor. ^‡^Please refer to Ref. 35 for details.

The DMLI examination has been designed by a DMLI examination committee composed of seven active ABMLI diplomates directing clinical immunology laboratories across a broad spectrum of professional spheres (academic medical center–associated clinical laboratories and a preclinical test development core, national reference laboratories, regional healthcare laboratories, and industry). A schematic representation of the pathway to and beyond the DMLI certification examination is depicted in Figure 1, and this also serves as a prelude to the remainder of the discussion in this report. The ABMLI examination was recognized by the CMS, and, similarly, in July 2023, the DMLI examination was approved by the CMS as a qualifying board for a high-complexity clinical laboratory director.

#### DMLI examination question vetting process

The ABMLI examination question bank, containing approximately 600 questions and kindly provided to the DMLI examination committee by the ASM, underwent a thorough, detailed, and item-by-item review by the DMLI examination committee to ascertain accuracy, clarity, currency, and relevance. Items that were deemed to be conceptually and/or factually outdated were removed from the question bank, and new items were developed by individual DMLI examination committee members on the basis of the current state of knowledge of human immunology, diagnostics, therapeutics, laboratory practices, and regulations. These new items were added to the question bank after being carefully reviewed collectively by the DMLI examination committee.

#### Standard setting for the DMLI examination

The DMLI examination will use the criterion reference standard where the examinees are evaluated against a set standard rather than against each other (norm reference standard) (54). The Angoff method was employed for the standard-setting procedure of the DMLI examination (55). The Angoff method is one of the most commonly used standard-setting methods and has been used for over 40 y in certification and licensure testing programs (55). For the standard-setting exercise of the DMLI examination, subject matter experts evaluated each examination question and judged the percentage of minimally competent practitioners who could answer the examination question correctly. Using the number 100 as a reference, subject matter experts provided ratings between 25% and 95% in increments of 5. After every expert’s ratings were collected, the grand mean of all ratings was calculated, and questions with outstanding SDs were flagged. Questions with large SD(s) (usually a benchmark of an SD of 10 but could be higher, depending on the examination) indicate a disagreement among experts that requires additional review and discussion. Whenever necessary, the experts reconvened to discuss and amend their ratings for the flagged items. After that, the grand mean was recalculated and served as the official cut score of the examination. The cut score is the minimum percentage of questions examinees need to answer correctly to pass the examination. For example, if the cut score is 65, examinees would need to answer at minimum 65% of the questions correctly to pass the examination.

#### DMLI examination format

This 4-h examination will contain 150 questions that will comprehensively evaluate both the breadth and depth of immunological knowledge of the test takers. The contents of the examination were directly informed by the results of a practice analysis survey sent out to 85 ABMLI diplomates, and the survey was carried out between July 27, 2020, and August 18, 2020, and was further guided by the input provided by the BOC committee. This practice analysis survey was carried out in accordance with the ASCP BOC policy and requirements of the accrediting body, the American (National Standards Institute) National Accreditation Board (ANAB), under International Organization for Standardization/International Electrotechnical Commission 17024. The practice analysis survey is a formal process for determining or verifying the responsibilities of individuals in the job/profession, the knowledge individuals must possess, and the skills and abilities necessary to perform the job at a minimally competent level (56). A practice analysis survey is required by psychometric standards, and it ensures that the certification examination is fair; valid; job related; and, most important, legally defensible (56). Broadly speaking, 15% of the DMLI examination will be focused on basic immunologic principles and mechanisms, 15% on immunologic techniques, 50% on immune diagnosis and clinical correlations, and 20% on laboratory management and operations. Several of the questions will involve case study scenarios that will test the examinee’s problem-solving and interpretive skills rather than simply direct recall of memorized facts. Hence, the ideal candidate seeking DMLI certification would have had formal graduate training in immunology (a doctoral degree) with relevant postdoctoral research and/or clinical immunology laboratory experience (Table I). This training should enable the candidate to get a firm grasp of the foundational, advanced, and emerging concepts across the entire breadth of the discipline. In addition, the candidate will require hands-on training in terms of clinical test development, on-call responsibilities, and understanding the nuances of clinical laboratory management as well as the regulatory aspects of directing a clinical laboratory (Table II). The DMLI examination committee has thoughtfully established well-defined guidelines and requirements that determine a candidate’s eligibility to take the examination (Table I) (30). With respect to route 1 (please refer to Table I), there are currently three Committee on Postgraduate Educational Programs (CPEP)-accredited programs in clinical laboratory immunology operating under the aegis of the ASM (57). The CPEP-accredited programs represent the most optimal training pathway in terms of the breadth and depth of the exposure to a clinical laboratory immunology curriculum the trainees need to master and gain expertise in (Table II) (57).

**Table I.** DMLI certification examination eligibility routes (current as of April 1, 2023)

	ASCP
Certification	DMLI
Education	Doctorate*^a^* from an accredited (regionally or nationally)*^a^* college/university, and
Training/experience	Route 1: Successful completion of a CPEP-approved program in medical laboratory immunology (dmli_training_doc_route_1.pdf [ascp.org]) or Route 2: 3 y of full-time acceptable postdoctoral training, research experience, and/or supervisory experience relevant to medical laboratory immunology in an acceptable laboratory*^a^* within the last 6 y. All experience must be completed postdoctorate (dmli_experience_doc_route_2.pdf [ascp.org]) or Route 3: 2 y of full-time acceptable predoctoral supervisory experience relevant to medical laboratory immunology in an acceptable laboratory,*^a^* and 2 y of full-time acceptable postdoctoral training, research experience, and/or supervisory experience relevant to medical laboratory immunology in an acceptable laboratory*^a^* within the last 5 y (dmli_experience_doc_route_3.pdf [ascp.org]).
Web site	https://www.ascp.org/content/board-of-certification/get-credentialed/#dmli

a Please refer to Web site for details.

**Table II.** CPEP immunology fellowship program curriculum essentials and guidelines (adapted from version 4, May 2022)

Major training areas	Essentials (minimum time to be spent in weeks)	Guidelines (range of time to be spent in weeks)
Infectious disease serology	8	8–12
Autoantibody testing	8	8–12
Histocompatibility testing and transplantation immunology	8	8–12
Innate and adaptive cellular immunology (flow cytometry, functional assays, etc.)*^a^*	8	8–12
Nonmalignant and malignant hematologic flow cytometry*^b^*	4	4
Immunodeficiency and other immune system disorders (evaluation of serum Ig levels, complement analyses, serum protein electrophoresis, etc.)*^a^*	4	4–8
Molecular biology/diagnostics	4	4–8
Public health serology	2	2
Soluble markers*^c^*	2	2
Allergy testing	2	2
Laboratory management and LIS/computer training*^d^*	8	8–12
On-call responsibilities*^e^*	12	≥12
Research	Open	
Teaching	Open	

a Immunodeficiency, other immune system disorders, monitoring therapeutic immune subset depletion and subsequent repletion, monitoring biologic therapies (mAbs) for immunologic disorders, evaluation of donor stem cell engraftment after hematopoietic stem cell transplant, and donor cell chimerism analyses.

b Examples include but are not limited to fetal hemoglobin analyses, evaluation of paroxysmal nocturnal hemoglobinuria, leukemias, and lymphomas.

c Examples include tumor markers (α-fetoprotein, human chorionic gonadotropin), cytokines, and chemokines. The trainees need to understand how certain cytokine (and soluble cytokine receptor levels such as soluble CD25) and chemokine levels correlate with disease course (for instance, in hemophagocytic lymphohistiocytosis) and/or response to therapy (e.g., during cytokine storm associated with chimeric Ag receptor T cell therapy and its management).

d Management includes handling issues related to laboratory safety, regulatory compliance, quality control and assurance, specimen collection, personnel management, clinical laboratory test coding and billing (Current Procedural Terminology codes), and laboratory workload assessment.

e Includes clinical consultation and direct communication with providers regarding laboratory results and troubleshooting laboratory test result–related issues.

LIS, laboratory information systems; laboratory informatics.

Each DMLI diplomate will be required to be recertified every 3 y. This recertification process will entail participation in 150 h of CE credits over the preceding 3 y and the payment of a recertification fee. Of these 150 h, 75 h will need to be category 1 credits as defined by the ASCP BOC (Table III) (30).

**Table III.** CE activities*^a^* (this is an adaption of the requirements established for recertification of ABMLI diplomates)*^b^*

**Category 1**
CE activities with accredited/approved sponsorship. For example, all activities that have approval by Professional Acknowledgment for Continuing Education (PACE), the Accreditation Council for Continuing Medical Education (ACCME), and the American Medical Association (AMA) for category 1 credit are approved. In addition, CE credit for the following activities can also be claimed under category 1: Authoring any full-length paper, short report, note, or case study published in a peer-reviewed journal (a maximum of three publications per recertification period can be claimed as category 1 credits). Any contribution to a textbook cataloged by the Library of Congress (a maximum of three contributions per recertification period can be claimed as category 1 credits). Preparing and presenting a lecture designated for AMA Physician’s Recognition Award category 1 credit or PACE credit. Achieving another doctoral-level specialty board certification. (The certification must be approved by the ACGME or CLIA.) Earning a medically related degree such as an M.P.H. Serving as an inspector for a CAP or New York State Laboratory Inspection. Writing evidence-based practice guidelines.
**Category 2**
CE activities without accredited/approved sponsorship (activities that do not satisfy the requirements for category 1, as described above). Examples include preparing and presenting a poster at a scientific meeting, authoring manuscripts and book chapters (if the number exceeds those claimed for category 1 credit), participation in National Institutes of Health study sections, writing a federal grant application (must be the principal investigator), and service on editorial boards, including ad hoc reviews.
**Category 3**
Teaching or lecturing about microbiology/immunology and/or career-related subjects (examples include university courses, review courses, and monthly didactic lectures).
**Category 4 (nonsupervised CE)**
Examples include review of self-instruction materials, taking self-assessment examinations, receiving education by a consultant, filing a patent, and serving on a scientific advisory board.

a For appropriate documentation required to verify these CE credit-earning activities and the CE hours that can be claimed for each activity, please contact the ASCP BOC.

b The recertification requirements for DMLI-certified individuals are currently a work in progress and will closely mirror the ABMLI recertification requirements.

ACGME, Accreditation Council for Graduate Medical Education; M.P.H., Masters In Public Health.

### The value of board certification in clinical laboratory immunology

In clinical medicine, board certification is the gold standard used to document a practitioner’s qualifications in a specific discipline. The U.S. Department of Health and Human Services and the 13 states that require licensure (California, Florida, Georgia, Hawaii, Louisiana, Montana, Nevada, New York, North Dakota, Rhode Island, Tennessee, West Virginia, and New Jersey) mandate that nonphysician clinical laboratory directors possessing a Ph.D. degree be board certified and/or possess credentials demonstrating appropriate competency to serve as a clinical laboratory director (28, 35). For many years, the ABMLI certification examination provided that documentation for doctoral-level immunologists (58). The ABMLI examination was discontinued in 2017, but the need to certify doctoral-level immunologists continues because board certification provides an important avenue for Ph.D. immunologists to direct clinical laboratories (28, 35). Unfortunately, training in immunology in general, and specifically cellular immunology (including but not limited to evaluation of subsets within CD4 and CD8 T cell and B cell populations, as well as cellular immune function), is not a major component of many Accreditation Council of Graduate Medical Education –accredited pathology residency programs. Therefore, the requirements, expectations, and duration of the training rotation may vary on the basis of the scope of immunology tests performed and/or available expertise of the faculty at the primary training institution. Hence, there is a critical need for appropriately trained and board-certified Ph.D.-level immunologists to direct high-complexity clinical immunology laboratories, particularly at academic medical centers. This need is only going to increase in the future because the field of medical laboratory immunology is rapidly advancing and encompasses many clinical subspecialties, including immune deficiency diseases, infectious diseases, autoimmunity, hematologic malignancies, allergy, and transplantation (12, 28).

At the very least, the training that is imparted to Ph.D. immunologists aspiring to become board-certified clinical immunology laboratory directors equips them with the necessary expertise and experience in four major facets of directing a high-complexity clinical laboratory:

1. The trainees learn how to rigorously evaluate the preanalytic, analytic, and postanalytic aspects of laboratory testing (59).
2. They acquire in-depth clinical knowledge of immunological diseases, which facilitates accurate, timely, and relevant testing of patients with these diseases, as well as the correct interpretation of test results based on the clinical context.
3. They also gain valuable firsthand experience in test validation and implementation, laboratory management, QC, QA, and understanding the regulatory mandates within which clinical laboratories operate (46, 60–62).
4. They are trained to recognize and execute the pivotal role the laboratory director plays in terms of laboratory stewardship. In this era of soaring healthcare costs, this function is an integral component of the laboratory director’s role. The laboratory director guides his or her clinical colleagues on the appropriateness of a given test and advises against unnecessary testing that may be expensive and does not add to the quality of care (63).

#### Other certification examinations with an immunology component available to PhD(s)

Since the termination of the ABMLI certification examination, individuals in need of board certification in medical laboratory immunology have been left with limited options. Of note, beginning in 1986, the American Board of Allergy and Immunology began offering a similar special certification in diagnostic laboratory immunology for practicing physicians; however, this special certification was terminated around the turn of the 21st century (64–69). There are currently three certification examinations recognized by the CMS/CLIA available to Ph.D.-level immunologists. These are the Bioanalyst Clinical Laboratory Director and the High-complexity Clinical Laboratory Director certifications offered by the American Board of Bioanalysis and a third certification offered by the American College of Histocompatibility and Immunogenetics (ACHI) (70–72). These three examinations vary quite widely in terms of the focus and emphasis on the breadth and depth of immunological knowledge that is evaluated. A major drawback of the High-complexity Clinical Laboratory Director and Bioanalyst Clinical Laboratory Director examinations is the 4-y training/experience requirement that must be met to sit for the examinations, and this limits the ability of individuals matriculated in the American Society for Microbiology (ASM)-affiliated, CPEP-accredited 2-y medical laboratory immunology fellowship programs to sit for the examination, even though they have the necessary training to successfully pass the examination (70, 71). The ACHI (formerly the American Board of Histocompatibility and Immunogenetics [ABHI]) offers the CMS/CLIA-recognized certification in clinical histocompatibility testing (72). This is a highly specialized examination, and, although it shares the coverage of administrative and technological aspects common to the broader field of diagnostic immunology, the clinical application is targeted to testing for solid organ and hematopoietic cell transplant. Thus, it only assesses competency and expertise across a limited component of the discipline of medical laboratory immunology.

### The value proposition of the ASCP brand

Laboratory medicine is estimated to contribute to 60–80% of medical decisions related to diagnosis and treatment (73), and, for the reasons outlined above, there is a growing need for board-certified nonphysician doctoral scientists to serve as directors of high-complexity clinical laboratories or clinical consultants in laboratory medicine or to work in research and development to develop new technologies and diagnostic tests (35). Currently, there is a paucity of appropriately trained board-certified clinical immunology laboratory directors (at present, there are only 81 active ABMLI diplomates, according to the registry maintained by the ASM).

The ASCP BOC is the credentialing agency that has developed and will offer the doctoral-level DMLI examination, credential, and credential maintenance program. The mission of the ASCP BOC is to provide excellence in certification of laboratory professionals on behalf of patients worldwide (29). The ASCP BOC is an independent, nonprofit certification agency governed by representatives from professional partners, as well as sponsoring, participating, and collaborating societies. While maintaining a corporate relationship with ASCP for fiscal and operational purposes, the BOC has autonomy in all governance and credentialing-related activities (29).

Initiated in 1928 as the ASCP Board of Registry, the BOC was formed by the merger of the ASCP Board of Registry and the National Credentialing Agency (29). To date, the BOC has credentialed over 625,000 laboratory professionals worldwide (29). The ASCP BOC certifies professionals who meet academic and clinical prerequisites and who achieve acceptable performance levels on examinations. With the addition of the DMLI examination, the ASCP BOC continues to credential laboratory professionals across the full spectrum of disciplines spanning clinical laboratory and pathology, from entry-level credentials through specialists, diplomates, and doctoral-level professionals (29).

Top indications for obtaining an ASCP BOC credential include evidence of professional competence and expertise, a strong sense of achievement, as well as improved capabilities for attaining a professional role and achieving higher compensation. Likewise, a sense of pride for recognized credibility fuels interest in attaining ASCP BOC credentials. Ultimately, attainment of professional credentials through the ASCP BOC is evidence of commitment to the profession and the patients we serve.

The ASCP BOC is the only certifying body for laboratory professionals in the United States accredited by ANAB (29). Full ANAB reaccreditation was again granted to the ASCP BOC, effective June 6, 2022, through June 7, 2027, per International Organization for Standardization/International Electrotechnical Commission 17024:2012 standards (29). ASCP BOC currently has accreditation for 22 certifications (29). The ASCP BOC is the sole provider of licensure examinations in the states of California and New York, and ASCP BOC credentials are recognized for licensure in all U.S. licensure states (29). Globally, the ASCP BOC has one of the most significant laboratory professional workforce certification and qualification portfolios, with 27 certification and qualification examinations worldwide (29). Often cited as the gold standard in global credentialing for medical laboratory professionals, the ASCP BOC is the first and largest credentialing agency (29). The ASCP BOC continues to elevate standards for quality and continuing competency while improving internal operation efficiency, advocating for the laboratory professional workforce, and aligning credentials with improved patient outcomes.

### Postcertification career paths

Career paths for those with appropriate certification in medical laboratory immunology are quite diverse, and these may be dictated by local, state, and government licensure requirements (Figure 1) (35, 74). For instance, currently, ABMLI certification is required by the New York Department of Health for doctorate-level laboratory directors overseeing moderate- to high-complexity immunology testing in several laboratories nationwide that are approved for testing samples from New York state residents (75, 76). A majority of ABMLI diplomates serve as directors of clinical immunology laboratories, whereas some diplomates who possess dual ABMLI and ACHI certification also serve as directors of histocompatibility and immunogenetics laboratories. Some of these laboratories exist at academic medical centers, but several operate in the nonacademic setting within larger regional or national reference testing laboratories (e.g., Quest Diagnostics and LabCorp). Opportunities also exist for certified medical laboratory immunologists who possess the necessary knowhow of clinical laboratory operations to lead clinical trials and direct translational research that can contribute to the development of (non–Food and Drug Administration–approved) clinical laboratory–developed tests. Companies that manufacture in vitro diagnostic products can also employ certified doctoral-level medical laboratory immunologists to serve as scientific or medical directors leading efforts in diagnostic immunoassay research and development to satisfy clinical and regulatory requirements. Last but not least, certified medical laboratory immunologists can also assume consultant roles at governmental entities such as the Centers for Disease Control and Prevention or the Food and Drug Administration. Below, we briefly describe a few of the more conventional career paths adopted by ABMLI diplomates.

#### Directing a clinical immunology laboratory in an academic setting

Overall, there are relatively few academic medical centers where the clinical immunology laboratory performs a broad phalanx of high-complexity immunology assays. Some of these laboratories are located at national reference laboratories affiliated with academic medical centers (e.g., Mayo Clinic Laboratories, the global reference laboratory affiliated with the Mayo Clinic; and the ARUP Laboratories, an enterprise of the University of Utah and its Department of Pathology), whereas the others exist at a limited number of freestanding children’s hospitals and some adult patient–centered hospitals that have active and well-established immunology and bone marrow transplant clinical divisions that provide care as well as curative treatments to patients with IEIs and other systemic disorders characterized by immune dysregulation. A major advantage of working at a national reference laboratory is that there is generally no dearth of human resources available for performing both the clinical testing as well as clinical test development. This allows a greater division of labor; however, because of the sheer scale of the operation existent at large reference laboratories, the individuals who direct the various laboratories within the immunology division can end up operating within scientific “silos” for which they serve as subject matter experts (e.g., allergy or autoimmunity or cellular immunology). Hence, such a milieu can potentially limit opportunities for broader scientific and administrative exposure and avenues for exploring new scientific directions. In marked contrast, a diagnostic immunology laboratory that operates within and serves a smaller hospital system is often led by a single director who oversees the full breadth of clinical immunology testing options, including IEIs, microbial and autoimmune serology, allergy and immunology, and hematologic malignancies. Moreover, in this setup, clinical consultative roles, oversight of quality, research, education, scientific literacy, community outreach, and regulatory compliance all fall within the bailiwick of a director’s responsibilities and core competencies. With the right training, mindset, and human resources, this is often an ideal ecosystem where free-flowing communication between the clinical providers and laboratorians speaking the same language (of immunology) focus their efforts on ensuring that the correct immunological tests are ordered and that the tests are performed at the highest level of expertise and correctly interpreted, which guides appropriate treatment decisions, and this ultimately directly benefits the patient.

In addition to the clinical consultative role, the director is also responsible for meeting or exceeding the established standards for quality control, quality assurance, and regulatory compliance. The director also oversees research and development activities in the laboratory aimed at bringing in or developing from scratch assays that advance the practice of clinical immunology. Furthermore, one can tailor one’s academic trajectory, depending on the domains of one’s faculty appointment, whether that is research (which entails being engaged in conventional bench research as well as applying for extramural funding), public health policy, or teaching geared at medical students, medical laboratory scientists, and clinical fellows.

#### Directing a clinical test development core

Optimizing therapeutics and diagnostics requires methodical developmental strategies for assuring efficacy, sensitivity, and reproducibility (77). Research and innovation are paramount in the pursuit of new treatments and diagnostic approaches aimed at individuals with a disease, and the translation of a potential diagnostic test or treatment option requires stepwise development from the bench to effective preclinical modeling and testing (78). In terms of laboratory diagnostics, the goal is to transition the innovation to a clinical validation step performed in a CLIA-approved laboratory, where it can directly benefit the patient (79). The initial development and preclinical testing are often done in a basic science laboratory and do not really require the foundational understanding of diagnostic or treatment translation to the clinic. However, when some of these new findings show great promise and strong potential for clinical application, basic scientists must reach out to certified and appropriately credentialed clinical laboratory scientists to perform the appropriate analytical and clinical validation studies before the test can be deployed in a clinical diagnostic laboratory (80). The profusion of diagnostic assays that currently assess biomarkers for various diseases is a great example of how the synergy between the clinical test development core and diagnostic laboratories can directly benefit patient care (81). In the right setting, if this pipeline operates as envisaged, this can be a symbiotic relationship in which the revenue generated by the clinical laboratory can also be used for sustaining the work and growth of the clinical test development core.

The initial discovery of a biomarker as a potential correlate of human disease subsequently requires an in-depth evaluation of assay performance characteristics, as described in *CLIA certification*, if it is destined to be a diagnostic test. If the discovery is intended as a therapeutic intervention, appropriate and rigorous preclinical testing has to be performed to evaluate the ideal therapeutic pharmacokinetics and efficacy to optimize the dose, minimize toxicity, and manage costs. Hence, directing an immunology clinical test development core requires a firm grasp of fundamental immunological concepts as well as the ability to discern and evaluate the clinical potential of a newly discovered immunological entity. Board certification in medical laboratory immunology helps broaden the scientific horizons and equips the individual to optimally evaluate both the scientific and clinically relevant pieces of the puzzle.

#### Directing a clinical immunology laboratory in a nonacademic setting

The scientific, clinical, and administrative responsibilities are not significantly different from those in an academic setting, but directing a laboratory that operates outside of the confines of academia is often a good fit for individuals who prefer to function relatively unencumbered by the expectations and responsibilities that accompany academic appointments, such as publishing, grant writing, and mandatory teaching. In many instances, such positions may also offer a greater degree of freedom and autonomy in terms of decisions relating to hiring laboratory personnel, test development, and validation. Additionally, if the laboratory is a standalone operation and has successfully established its niche, there might be fewer financial constraints to contend with as opposed to laboratories operating within a hospital, where there is greater competition for getting a piece of the financial pie allocated to laboratory operations.

## Conclusions

Given the current trajectory of immunology as a scientific discipline and its ever-increasing relevance to multiple branches of clinical medicine, we believe that the ASCP BOC represents the right home for the medical laboratory immunology certification examination.

Our hope and vision is to spread awareness about the DMLI certification examination within the immunology community and spotlight the important role that doctoral-level (including Ph.D.-trained) board-certified clinical laboratory immunology directors play in the day-to-day operations of modern healthcare delivery. We sincerely wish that this report will spark considerable interest especially among immunology graduate students and postdoctoral fellows and that these individuals might seriously consider clinical laboratory immunology as a fulfilling and rewarding career path where their scientific and intellectual skills can be put to good use.
